# A colonial-nesting seabird shows no heart-rate response to drone-based population surveys

**DOI:** 10.1038/s41598-022-22492-7

**Published:** 2022-11-05

**Authors:** Erica A. Geldart, Andrew F. Barnas, Christina A. D. Semeniuk, H. Grant Gilchrist, Christopher M. Harris, Oliver P. Love

**Affiliations:** 1 Great Lakes Institute for Environmental Research, University of Windsor, Windsor, ON Canada; 2 Department of Integrative Biology, University of Windsor, Windsor, ON Canada; 3 National Wildlife Research Center, Environment and Climate Change Canada, Ottawa, ON Canada

**Keywords:** Animal physiology, Ecophysiology

## Abstract

Aerial drones are increasingly being used as tools for ecological research and wildlife monitoring in hard-to-access study systems, such as in studies of colonial-nesting birds. Despite their many advantages over traditional survey methods, there remains concerns about possible disturbance effects that standard drone survey protocols may have on bird colonies. There is a particular gap in the study of their influence on physiological measures of stress. We measured heart rates of incubating female common eider ducks (*Somateria mollissima*) to determine whether our drone-based population survey affected them. To do so, we used heart-rate recorders placed in nests to quantify their heart rate in response to a quadcopter drone flying transects 30 m above the nesting colony. Eider heart rate did not change from baseline (measured in the absence of drone survey flights) by a drone flying at a fixed altitude and varying horizontal distances from the bird. Our findings suggest that carefully planned drone-based surveys of focal species have the potential to be carried out without causing physiological impacts among colonial-nesting eiders.

## Introduction

Accurate, consistent, and economical surveys of wildlife populations continue to be a fundamental element supporting wildlife science and management. Aerial imagery techniques have been traditionally used to detect and count individuals to derive a range of demographic metrics including population size estimates^1–9^, spatial distribution^10,11^, temporal and spatial dynamics of colony formation^12^, operational sex ratios^13^, nest survival estimates^14^, and fine-scale foraging behaviours^15–17^. Traditional methods for censusing colonial-nesting bird populations and nesting distributions have included ground-based and occupied aircraft surveys, as well as remote sensing techniques like satellite imagery^18^. Ground-based surveys gather data at smaller spatial scales to monitor smaller or more cryptic species, but can be time consuming. Further, viewing nests from a great distance can be difficult, therefore data collection on the ground may require close-proximity of researchers to individual nests^19^. By contrast, occupied aerial surveys are designed to rapidly cover large areas, but can be logistically difficult, expensive, and dangerous when conducted in remote areas^20–22^. Importantly, both investigator intrusions and aircraft activity can induce changes in nesting bird physiology and behaviour, suggesting disturbance effects can be associated with both techniques^23,24^. Finally, while commercial satellite imagery is promising for wildlife surveys due to broad spatial coverage and lack of investigator disturbance, such imagery is too low resolution for identifying individuals and may be restrictive in polar regions due to cloud cover^6,7,25^.

There are now a number of emerging techniques used to survey wildlife, including the use of aerial drones^26^. Drones can collect large quantities of data, be flown in inaccessible locations^5^, are thought to mitigate disturbance^3,19^ and can provide accurate, precise, and consistent population estimates^4,19,27,28^. Despite the apparent advantages, the use of drone technology in the study of sensitive species such as colonial birds has raised concerns about their disturbance effects to nesting birds^19,29–32^. Many of the studies that aim to quantify the effects of drone disturbance on wildlife have been explicitly designed to use the drone as a disturbance stimulus to test whether responses are proportional to the distance (e.g., lateral distance or survey altitude) between the stimulus and the responder^29–32^, or whether different flight patterns (e.g., “target-oriented”, “lawn-mower”, and “hobby”^33^) elicit different responses^19^. While preliminary investigations of drones as disturbance stimuli are informative for understanding the behavioural responses of birds, these studies are often designed and executed in ways that may not necessarily reflect standard survey methodologies that researchers would implement in the field. In such scenarios, the experimental response of birds to an approaching drone aircraft may not be representative of a response observed during a formal survey design^34^.

To date, research on the effects of drones on birds has focused primarily on behavioural alterations of colonial-nesting individuals; e.g.,^19,30,31,35^, without considering the potential physiological responses that may occur even when behavioural changes are not observed (e.g.,^36^). Breeding birds are often highly attached to their nest during the incubation period, and reluctant to leave to ensure their breeding success^37^. Consequently, studies that measure behavioural responses such as nest abandonment as the sole indicator of stress may overlook the possibility that physiological stress responses are occurring but are undetected (e.g., increases in stress hormones or heart rate). Thus, studies that only examine behavioural responses may not fully capture the potential impact that drones may have on their study subject(s)^32,38^. Of the few studies that have assessed a physiological metric of stress^38,39^, only one has focused on birds^32^.

Hormonal responses (e.g., circulating glucocorticoids) are a commonly used measure of physiological stress in field studies; e.g.,^40,41^, and can be reliable predictors of stress-responsiveness in birds^42^. However, the collection of blood samples requires the capture and handling of free-living birds which itself, has the potential to interrupt incubation and chick-rearing behaviours of the focal bird and even those around them; e.g.,^43^. More recently, metrics such as the measurement of heart-rate responses during stressful events has been used as real-time physiological measures of an animal’s assessment of a stimulus in the field^44–48^.

To the best of our knowledge, the only study to investigate avian heart-rate responses to drone flights was conducted by Weimerskirch et al.^32^, which involved intentionally flying a drone over adult and chick King penguins (*Aptenodytes patagonicus*) to examine their responses to drone passage and approaches using externally mounted heart-rate biologgers. The authors found that both adult and chick heart rates increased in response to drones at low altitude (2–50 m above ground) surveys by the aircraft. While these results demonstrate the potential for approaching drones to cause physiological stress responses in birds, there is still a need to estimate these responses during standardized transect surveys (i.e., species-specific drone methods that would actually be used to collect imagery of nesting colonies).

**Figure 1:**
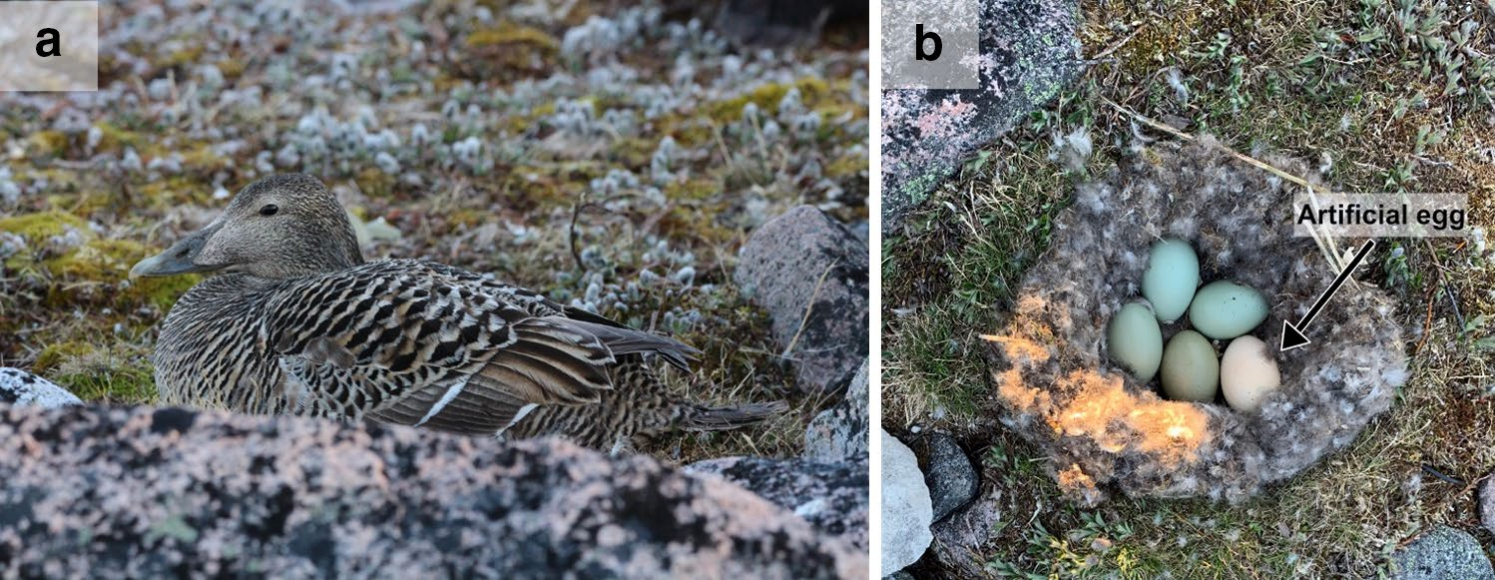
Photographs of (**a**) an incubating female Common eider *Somateria mollissima* (photo credit to Erica Geldart) and (**b**) an artificial-egg (white egg) in a Common eider nest (photo credit to Reyd Smith).

We investigated changes in heart rate of incubating female Common eiders (*Somateria mollissima*, hereafter, “eiders”) in response to colony survey flights by a small, quadrotor aerial drone. Since female eiders are highly cryptic during incubation (Fig. 1a) and nest densely within easily-disturbed colonies where ground-based surveys are impractical^49^, drone-based surveys offer an efficient method to accurately survey population numbers and densities, as long as they do not unduly disturb birds. Rather than using a drone to test at which specific height or speed eiders responded physiologically to a drone, we instead collected data on birds equipped with heart-rate monitors during an actual transect-based drone survey that was carried out to quantify nesting eiders. We proceeded with the hypothesis that incubating eiders would interpret the flight of a survey drone as an environmental stressor, and that eiders would exhibit an increase in their heart rates during drone surveys as compared to their resting, baseline heart rates measured in the absence of drone survey flights. Our overall goal was to quantify whether aerial drone flights elicited a physiological response by nesting eider ducks, and assess whether drone aerial surveys are a practical method for surveying colonial birds non-invasively.

## Results

The two drone flights required to map the entire study area at 30 m Above Ground Level (AGL) took 43 min, with an approximately 4-min-long gap in between flights to change batteries and SD cards. During drone flights, no mammalian predators (e.g., Polar bears *Ursus maritimus*, or Arctic foxes *Vulpes lagopus*) were present on East Bay Island. Nonetheless, Herring gulls *Larus argentatus* are common on East Bay Island (i.e., 25–30 breeding pairs annually) and were therefore present during both flights. No focal eiders left their nest during drone flights (i.e., displayed a flush response), and none of our focal nests were predated during the study period.

We collected 93 heart-rate samples from 11 focal eiders across the four drone distance categories. Mean date of incubation (DOI) on June 29th was 7.6 days (± 4.0 SD). For most individuals, we were able to collect 2 to 3 samples for each distance category, although three birds had categories where we were unable to collect a sample of sufficient audio quality (see Table S1 in Supplementary Materials).

**Figure 2:**
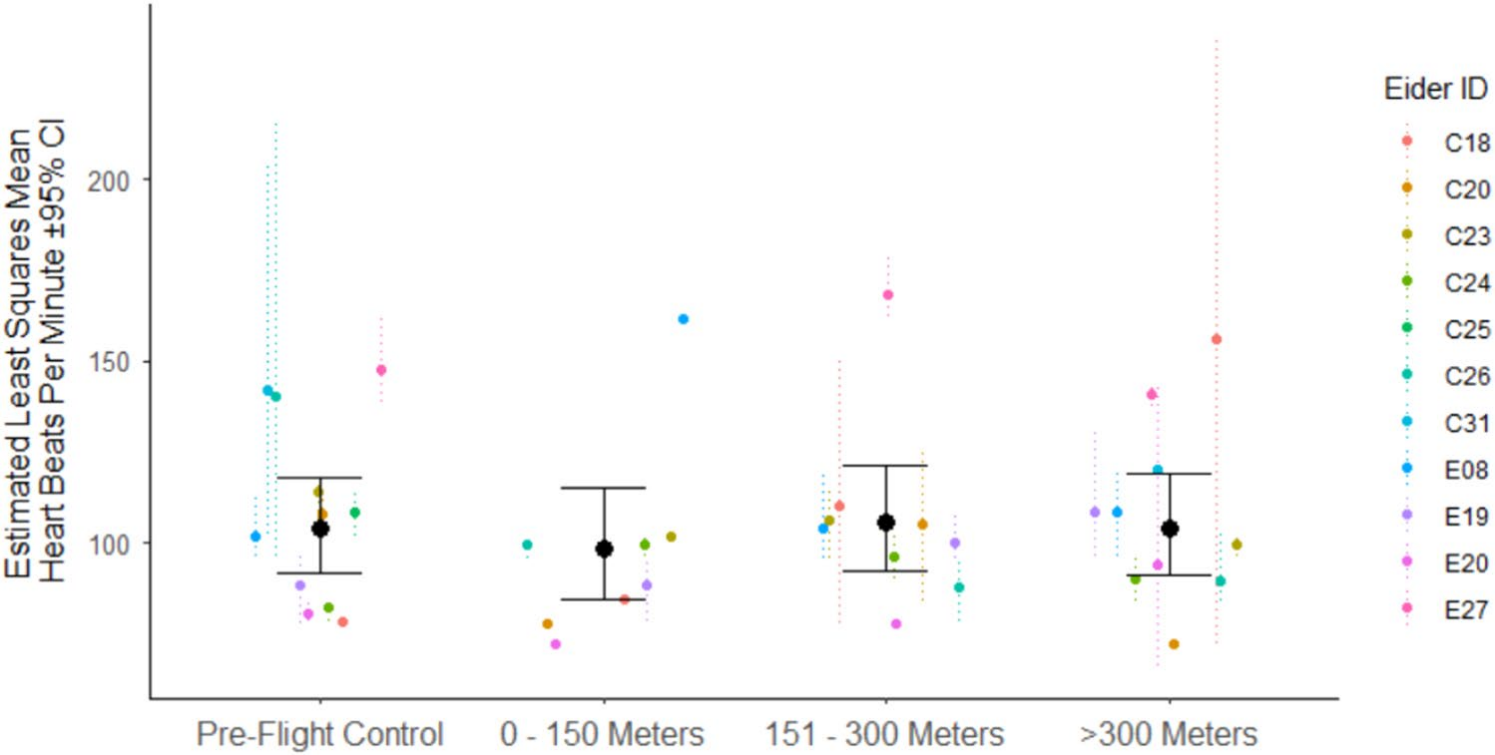
Data scale model estimates for Common eider (*Somateria mollissima*) mean heart rates (bpm) in each distance category (± 95% confidence intervals). Dashed, coloured lines indicate mean and range of raw heart-rate data for individual eiders. Note that points without range bars indicate only a single data point for that individual within that category. Ranges jittered for clarity.

We failed to detect a statistically significant difference in heart-rate response across all categorical treatment levels (control, 0–150 m, 151–300 m, > 300 m; F_3,58_ = 0.31, *P* = 0.82, Table 1). We also failed to detect a statistically significant difference in heart-rate response due to DOI (F_1,58_ = 0.01, *P* = 0.91). These results suggest that incubating eiders within our study did not show a change in heart rate as a result of our drone-based population survey, while accounting for DOI. Concordantly, least means data scale estimates of the mean and confidence intervals were similar for each distance category (Fig. 2). Our random effects structure significantly improved model fit (LRT: *χ*^2^(2) = 16.34, *P* < 0.0001), and covariance estimates for eider ID were higher than sample number (i.e., 10-s heart-rate samples within each distance bin). This indicates that there was a higher proportion of variance in heart rate attributed to individual eider, rather than sample number (i.e., intra-individual variance). Overall, our full model, that included our distance categories and DOI as fixed effects, received less support than the intercept-only model (AICc = 21.40 vs. 4.18 respectively).

**Table 1:** Model parameter estimates for effect of drone distance category (controlling for reproductive investment; DOI – date of incubation) on heart rates of nesting Common eiders (*Somateria mollissima*).

Model parameter	Estimate ± SE
**Fixed effects**	
Intercept	4.658 ± 0.13
0–150 m	− 0.056 ± 0.07
151–300 m	0.014 ± 0.06
> 300 m	− 0.001 ± 0.06
DOI	− 0.002 ± 0.01
**Covariance estimates**	
Eider ID	0.027 ± 0.02
Sample number^¥^	0.0003 ± 0.005
Residual	0.047 ± 0.008

Estimates obtained from 93 observations across 11 individual eiders. Distance categories are compared to reference category that is the control period before drone flights occurred.

^¥^Sample number nested within Eider ID.

## Discussion

The highly cryptic camouflage of incubating female eiders makes them particularly difficult to survey. To detect nesting eiders using aerial photography necessitated that aerial drones fly 30 m from the ground and fly quickly at approximately 10 m^−s^ (Jagielski, Love and Semeniuk, unpubl. data); both requirements having the potential to disturb nesting eiders. Despite this, results from our study indicated that these drone-based survey methods did not impact nesting female eider heart rates and suggests that drone imagery can be used to survey nesting eider ducks; a species known to be sensitive to ground-based surveys^50^. Our study therefore adds to the growing body of literature which suggests that, if used appropriately, drones can be a suitable tool for surveying wildlife populations while minimizing disturbance (e.g., Adélie penguins *Pygoscelis adelia*, Lesser black-backed gulls *Larus fuscus*, and several species of penguin, albatross, and cormorant^3,30,32^). However, as the eiders in our study had been previously exposed to practice flights and other drone projects on the island earlier in the season (see “Methods”), it is possible that some degree of habituation occurred, masking any novel-stimulus response. However, we believe that sensitization to repeated flights is unlikely because of our lack of response despite our eiders being exposed to previous flights. We acknowledge this logistical difficulty, but contend that our findings still hold merit as an initial examination of heart-rate response to this emerging wildlife survey tool. In future studies, we recommend researchers explore whether heart-rate responses vary over successive flights. Here we discuss our findings in relation to other relevant survey protocols used within and across breeding bird populations, as well as implications of our findings for the long-term monitoring of eider ducks. Note that the effect of recreational drone use is beyond the scope of this study and the results should not be interpreted as such.

Our findings suggest that drone surveys offer a less invasive alternative to ground- and occupied aircraft-based surveys, which have been associated with increases in heart rate by colonial-nesting seabirds (e.g., Adélie penguins, Wandering albatrosses *Diomedea exulans*, Giant petrels *Macronectes halli*^36,46,51,52^). The only study (to the best of our knowledge) that has directly compared avian responses to multiple censusing methods found that drones caused less disturbance in terms of behavioural alterations, compared to investigators walking through the colony making counts (e.g., Lesser black-backed gull^3^). However, a lack of behavioural response is not indicative of no response, e.g.,^36^; thus additional studies that use heart rate as a physiological metric for disturbance to directly compare animal responses across different censusing methods are needed.

We found that incubating eiders did not leave their nest, nor alter their heart rate, in response to a rotary propeller drone flying over them as part of grid-pattern survey flights. Nonetheless, we caution that this lack of heart-rate response at the tested altitude should not be considered repeatable in other species without further testing. For example, the perceived level of threat generated by a drone can depend on both the attributes of the drone and modes of operation^33^. Our study area is relatively small and can be mapped efficiently with slower moving rotary-wing drone models (Jagielski, Love, and Semeniuk, unpubl. data). Alternatively, fixed-winged aircrafts, which may be better suited to large-scale research on non-cryptic species, may resemble the silhouettes of many avian predators, resulting in a higher perceived threat by bird species compared to quadcopter-style drones^33,53,54^. The size of the drone is also known to influence animal responses, with larger drones causing greater reactivity than smaller ones^33^. Moreover, the noise level of a multirotor drone is limited when compared to fixed-wing and fuel-powered drone^55^. Whether a species can detect the sound emitted by a drone and at what specific threshold drones cause acoustic disturbance depends on ambient noise levels, auditory capabilities of target species, and drone altitude^55,56^. For example, the noise emitted by a multirotor drone of similar size class and at the same altitude as in the current study was lost in the background noise and therefore caused no disturbance to Chinstrap penguins (*Pygoscelis antarcticus*) during breeding^55^. Moreover, research supports the ‘distance hypothesis’, which posits that animals display fewer disturbance responses to drone flights at greater altitudes^29,32,33^. As such, disturbance impacts from the use of larger, noisier, or fixed-wing drones in other study systems may be mitigated by the comparatively higher survey altitudes typical of these models^30^. Research also indicates birds are more likely to respond to drones exhibiting flight patterns similar to those used by avian predators such as vertical flight patterns and target-oriented approach angles rather than horizontal flight patterns or tangential angles^33,54,57–59^. Although our population-census work found no impacts on heart rate, future planned flights at different altitudes and patterns may be required in future eider studies. As such, further testing of whether those altered flight techniques impact physiological responses in eiders may be justified. Importantly, we recommend these future studies be designed to examine disturbance effects in the context of actual survey protocols that would be used for data collection in respective study systems instead of experimenting with drone-based methods that would not be reproduced in standard drone-use protocols.

A bird’s perceived level of threat by a drone can also depend on characteristics of the birds themselves^33^. Birds may respond differently to disturbance depending on the investment ‘value’ of their current clutch. Birds often exhibit greater behavioural (e.g., flushing distance by several species of waterfowl^60^; return time by Common eiders^49^) and physiological responsiveness (e.g., greater modulation of heart rate by King penguins^61^) to disturbance in their earlier stages of breeding than birds further along in their breeding, presumably because the probability of eggs surviving until hatching increases and so does the expected benefit of current reproduction relative to the parent’s survival and future reproduction^62^. The response of eiders to drone flights in this study did not differ in relation to their stage of incubation up until 17 days. We note that our research did not evaluate the response of eiders during the last stage of incubation, when body lipids are depleted (i.e., phase III at 23–26 days of fasting^63^). Because stress-induced heart-rate responses can be energetically costly for birds, e.g.,^64^, we might expect the largest effect of incubation stage on eider responses to occur at the end of their incubation period when eiders are most energetically limited. Given the timing of our study in relation to eider incubation stage (see “Methods”), we cannot evaluate this possibility. As such, drone surveys of eiders occurring during the final phase of incubation and fasting should be studied, particularly if a project intends to carry out repeated flights throughout the breeding period (e.g., colony formation mapping^12^). Next, individual variation in circulating corticosterone may differentially mediate physiological and behavioural responses to threats, e.g.,^41,65^, and may therefore create inter-eider variation in sensitivity to drones. Finally, for nesting eiders in our study area, the threat of avian predators comes primarily from herring gulls that commonly consume eider eggs and ducklings, but pose little threat to adult eiders. However, other breeding populations of eiders or avian species that experience predation from avian predators of adults may perceive a greater threat to drones.

Research efforts on drone technology and best practices for wildlife and ecological science are still developing as a methodology, so efforts must be made to minimize disturbance to focal wildlife^66^. For example, human disturbance can produce long-term effects on colonial breeding bird individuals and populations^23,24^. Incubation alone has a high energetic demand for eiders^67,68^, so any further energy loss from drone disturbance during the incubation period may have negative reproductive fitness consequences. Findings from the current study suggest that eiders do not endure fitness consequences associated with a heart-rate response from a drone flying over them during a transect survey design. Research that monitors both behavioural and physiological responses of wildlife during comprehensive drone-based survey methods will help improve population survey results, as well as evaluate the impacts of drone use for wildlife studies, and ultimately help to minimize disturbance to wildlife^69^.

## Methods

### Study species and area

**Figure 3:**
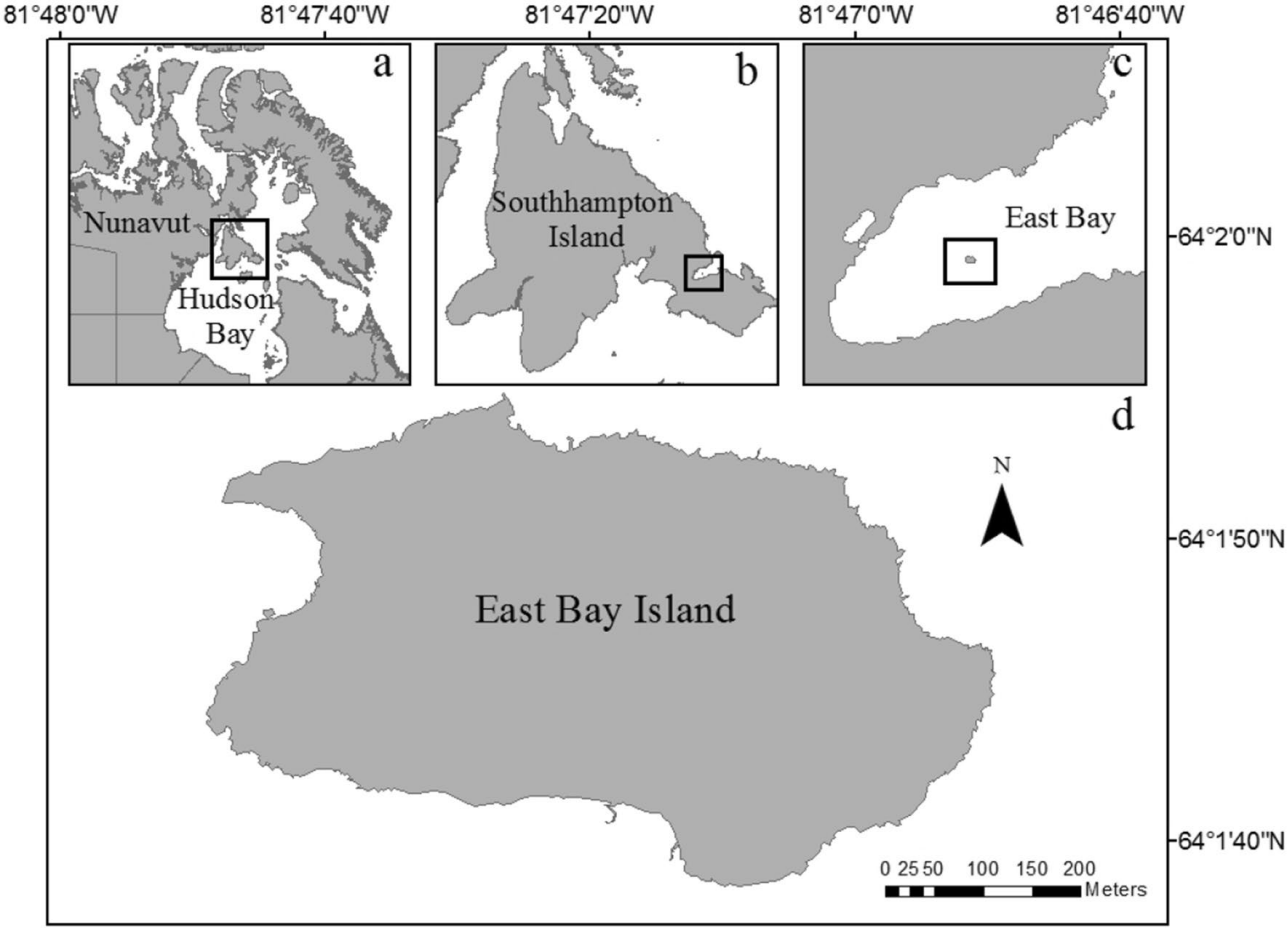
Map of study area, (**a**) Nunavut, Canada, (**b**) Southampton Island, Nunavut, Canada, (**c**) East Bay, Nunavut, Canada and (**d**) East Bay Island, Nunavut, Canada. Canadian Provinces and Territories map layers provided by ESRI online, accessed May 30, 2018. Map layer of East Bay Island created using ArcMap v10.6.1 (Esri, Redlands, CA, USA).

We conducted our study on incubating female eiders nesting on East Bay (Mitivik) Island, Nunavut, Canada (64° 02′ N, 81° 47′ W) in June of 2019. East Bay Island is a small (approximately 800 × 400 m), low-lying, and predominantly rocky island (Fig. 3) that supports the largest known Common eider breeding colony in the Canadian Arctic (Inuit Nunangat)^70^. Breeding and demographic studies on this colony have been conducted continuously since 1997^50,71–73^. Traditionally, breeding colony size and density estimates have been derived via multiple methods; namely, trained observers in fixed observation blinds have conducted point counts of female eiders nesting in long-term study plots and scaled up to the area of the island, as well as hatched eider nests at the end of the breeding season (Love et al. pers. comm.). However, although these estimates have been robust, they are inherently associated with a degree of uncertainty. Moreover, over the past 10 years, factors such as large-scale mortality from novel disease outbreaks (e.g., avian cholera^74–76^) and an increasing rate of Polar bear nest predation^77,78^ has made quantifying accurate estimates of colony size and density increasingly difficult and dangerous to collect in this manner. As such, we have begun to turn to aerial drone surveys to determine whether the dynamic breeding colony can be censused safely and more accurately than via previous methods. No animals were handled for the current study.

### Heart-rate monitoring

**Figure 4:**
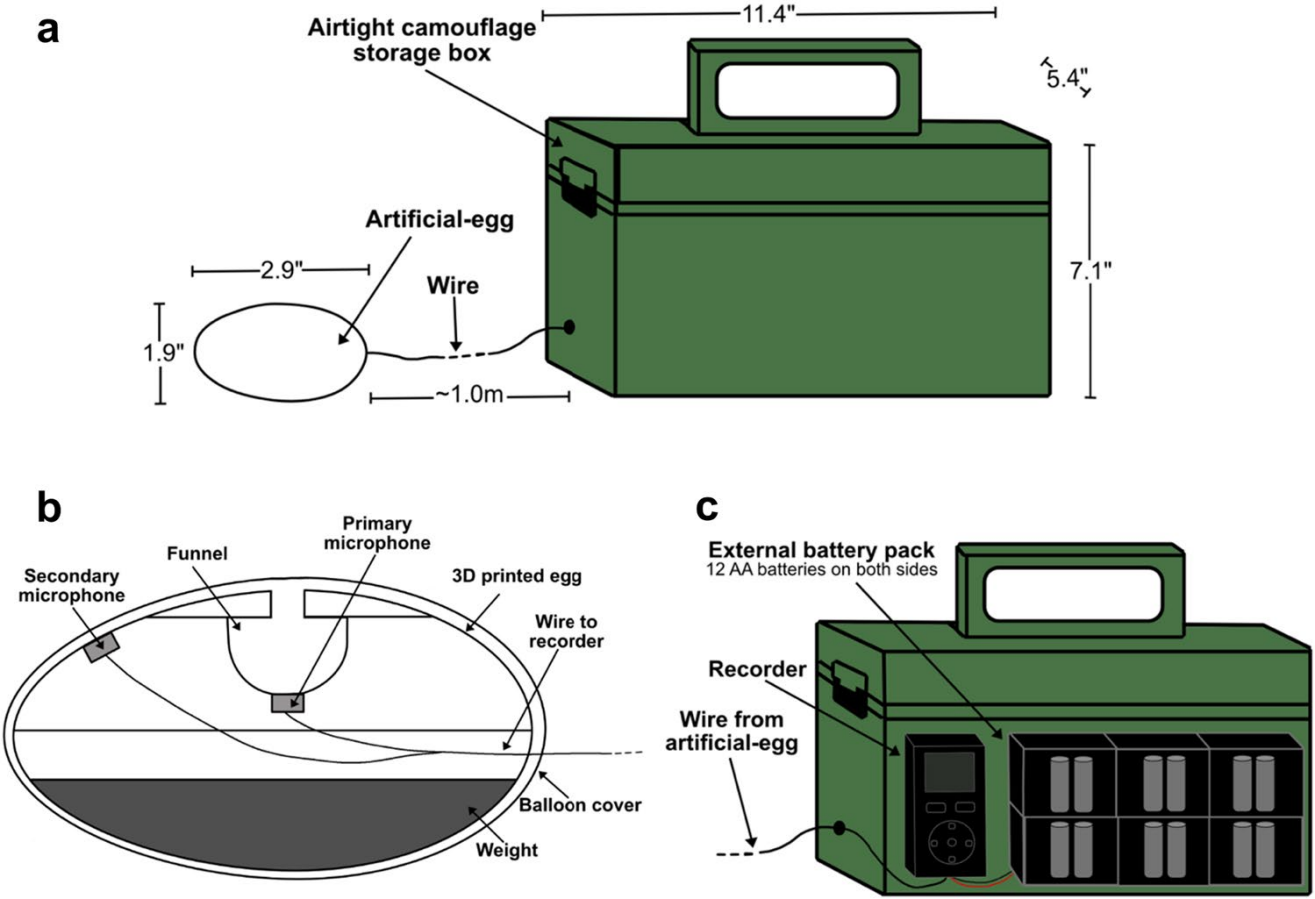
Schematics of (**a**) an artificial-egg heart-rate monitor, (**b**) the interior of the artificial-egg and (**c**) the interior of the airtight camouflage storage box.

On June 24th, 25th and 28th, we deployed heart-rate monitoring equipment during laying/early incubation in active eider nests (n = 11) for another set of projects (Geldart et al. in review). Study nests were located in areas of low nesting densities to limit researcher-induced disturbance in denser portions of the colony. Study nests were each located an average (± SD) of 302 ± 162 m (range: 11–560 m) apart from each other. Each nest was equipped with an artificial-egg heart-rate monitor. Our heart-rate monitors were adapted from Giese et al.^79^, who found that resting heart rate of Adélie penguins recorded using artificial eggs were indistinguishable from those recorded using electrocardiogram units attached externally to the penguins (Fig. 4; see Geldart et al. in review for more details). Heart-rate monitors (Fig. 4a) consisted of a 3D-printed plastic eider egg (sub-elliptical, 2.9 in. long × 1.9 in. at the widest point, Fig. 4b) equipped with two Electret condenser microphones (PUI Audio model AOM-5024L-HD-R) for stereo recording. The microphones were each soldered to the bare end of a shielded cable assembly (approx. 72 in. long) with a stereo plug (3.5 mm) at the opposite end. The primary microphone was situated at the end of a 3D-printed plastic funnel for amplified sound while the secondary one was pressed against an opening on the surface of the egg to increase the likelihood of recording heart sound when the eider shifted positions on the egg (i.e., off the primary microphone). We weighted the bottom half of the egg to ensure the egg maintained a fixed orientation in the nest, with the microphones always facing toward the eiders’ brood patch. Once equipment was assembled, each half of the 3D-printed egg was fastened together with glue and covered by a balloon membrane for waterproofing. Both microphones were wired to a recorder (Tascam DR-05X equipped with a 128 GB microSD card) by plugging the stereo plug into the recorder’s stereo mini jack. The recorder was also attached to an external assembled battery pack (with 24 AA Lithium Ion batteries) to allow for 11–12 days of continuous recording. The recorder and battery pack were situated within a weatherproof camouflaged storage box (11.4 in. long, 5.4 in. wide, 7.1 in. high, Fig. 4c) located approximately one meter outside the nest and the box and cable were secured with the surrounding terrain. The first-laid egg from each study nest was collected and used to estimate incubation stage (see Estimating incubation stage below). This first-laid egg was replaced with the artificial egg (Fig. 1b). Previous research has suggested that using artificial-egg heart-rate monitors had no effects on incubation behaviour^79^ or nest survival^47^, thus it was assumed that incubating the artificial eggs would not affect the eiders differently from incubating their natural eggs. In the current study, no nest abandonment occurred after after nest equipment deployment and birds returned to their nest approximately 71 min (i.e., average, 5 s to 7 h range) after equipment was deployed on their nest.

### Estimating incubation stage

The first-laid eggs collected from each study nest were immediately candled to assess the stage of embryo development and hence to estimate the DOI for each focal hen^80^. These methods provided estimates of the number of days the first-laid eggs had been incubated on the day heart-rate monitoring equipment was deployed, and therefore the degree of incubation investment females had made up to June 29th (i.e., the day of drone survey flights). DOI was measured as a means to control for potential changes in heart rate with increased investment in the clutch^61^.

### Drone surveys

We conducted drone survey flights on June 29th using a small, quadrotor DJI Phantom 4 Pro drone. However, prior to these flights on June 29th, several practice flights and other drone projects had taken place on the island. Thus, the flights investigated on the 29th were not the only flights to which eiders had been exposed, but these were the earliest flights available to which we could pair heart-rate recorders to. Flights were planned as semi-autonomous parallel line transects at 30 m AGL. Survey altitude and flight paths were chosen to produce a georeferenced orthomosaic of sufficiently high image resolution to estimate the number of nesting eiders on East Bay Island. Due to battery life constraints, two survey flights were required to map the entire 24-hectare island at 30 m AGL. Briefly, the drone was launched from the East Bay Island research compound, and the drone automatically moved towards the start of its line transect, where it then moved along a straight-line flight path collecting imagery. Following completion of each transect, the drone moved to the next transect start position and continued with this “lawn-mower” flight pattern^33^ until the end of the flight. The two drone flights conducted on June 29th took place from 8:52:37–9:13:12 and 9:17:28–9:35:59.

During surveys, the drone’s position (latitude and longitude) was logged several times each second with an associated Unix timestamp, stored onboard the drone’s SD card which were downloaded following flight completion. We used this time and position data for the drone to estimate distance of the drone to each focal eider nest throughout each flight. We paired this time and location data of the drone with the eider heart-rate monitors to estimate heart rate of eiders for different distances to the drone during surveys (see Heart-rate quantification). For additional technical specifications and details on flight operations, see the Drone Reporting Protocol in Supplementary Materials^81^.

### Heart-rate quantification

To quantify eider responses to our drone flight survey, we made opportunistic use of the heart-rate recording data. We reviewed heart-rate recordings of eiders using the sound analysis software Audacity (Version 2.3.2)^82^. We synchronized drone GPS and heart-rate monitors to the nearest minute from the clock of an iPad (Apple Inc.). We estimated baseline (i.e., resting) heart rate of eiders by collecting heart-rate samples approximately 2 h before drone flights commenced. We collected up to three 10-s samples from each eider at approximately 7:00am, 7:15am, and 7:30am on June 29th. No researchers were present on the ground within the eider colony during the study period, but instead were all working within the research compound (approx. 12 m from the nearest eider nest and not within view of the incubating eiders). For measures of heart rate during the drone flights, the lateral distance of the drone from each nest was separated into three distance categories (0–150 m, 151–300 m, and greater than 300 m). We chose these large distance categories due to the relatively fast flight speed of the drone (approximately 10 m^-s^). To accommodate any differences in temporal resolution between the drone and the heart-rate monitors, we identified time periods where the drone was within a given distance category for ≥ 60 s, and collected 10-s samples of heart rate from the approximate center of that time period^48^. For example, if a drone was within 0–150 m of the focal eider nest from 08:55:30 to 08:56:30, we attempted to find a clean audio sample from the heart-rate recorder at the approximate 08:56:00 mark in Audacity. To maximize our sample size, we sometimes shifted our sampling time several seconds to obtain unambiguous heart-rate measures. Unclear recordings may result from the bird moving on their nest, which has been associated with increased heart rate^83^, thus avoiding unclear recordings of heart rate may skew our data. However, discarding ambiguous measures of heart rate (as done in previous studies using artificial-egg heart-rate monitors^47,48^) would have the same effect and reduce sample size. For each focal nest, we collected up to three heart-rate samples in each distance bin along with the control period before the drone flight started. However, not all focal nests had clean audio files for each distance bin, resulting in different sample sizes for each bird in each bin (see Results). We extracted all the samples as wav files and heartbeat sounds were counted aurally at least twice to avoid measurement error and therefore increase accuracy of the counts. Finally, we extrapolated each 10-s sample to beats-per-minute for statistical analysis of heart rate.

To confirm eider presence on their nest during drone flights we combined the heart-rate recording data with simultaneous video footage recorded from Browning trail cameras (model: BTC-5HDPX, set to motion-trigger activation) placed approximately one meter from each focal nest for another project (Geldart et al. in review).

### Statistical methods

To estimate changes in eider heart rate (bpm) during drone surveys, we constructed linear mixed models using PROC GLIMMIX in SAS Studio v3.8^84^. We modeled eider heart rate as a function of the fixed effect for distance category (categorical with 4 levels: control period before flight, 0–150 m, 151–300 m, > 300 m), as well as a fixed effect for estimated DOI on June 29th (continuous, range 3–17). We included heart-rate sample number as a nested random effect for each distance category within eider ID. Since sample numbers for each distance category were ordered by time of day (e.g., sample 1 comes before sample 2, etc.), this nested random effect structure should partially account for the increased exposure of eiders to the drone throughout the sampling period. We tested whether this random effect structure significantly improved model fit compared to the fixed effect only model using a likelihood ratio test. We log-transformed eider heart rate to facilitate the assumption of a Gaussian distribution, assumed a variance-components covariance structure, and an identity link function. Note that we intentionally did not include an autoregressive covariance structure here as (1) exploratory autocorrelation function plots did not reveal any obvious signs of temporal autocorrelation, and (2) individual samples for each eider were not equally spaced in time due to the opportunistic nature of heart-rate data collection, which violates a main assumption of the autoregressive covariance structure. We assessed model fit by examining Studentized and Conditional Pearson’s residual plots and comparison of AICc scores between our full model and an intercept-only model^85,86^.

We back transformed model least-square means and 95% confidence intervals of the mean to the original data scale (heart rate bpm). Prior to model construction in SAS, all data manipulation was done with packages *dplyr*^87^, *lubridate*^88^, and *geosphere*^89^, while plots were constructed with *ggplot2*^90^ in R Studio v3.6.2^91^. For all statistical significance tests, we used α = 0.05.

### Permit statement

Data collection and monitoring of eider nests were authorized by Migratory Bird Sanctuary Permit, Canadian Wildlife Service MM-NR-2019-NU-013, Migratory Bird Scientific Permit, Canadian Wildlife Service SC-NR-2019-NU-006, Water Licence, Nunavut Water Board 8WLC-PCE1920, Wildlife Research Permit, NU – Department of Environment – Wildlife Division, Land Use Permit, Indigenous Affairs and Northern Development Canada, Animal Care Permit, Environment and Climate Change Canada 19GG26, EC PN 18 026, Animal Care Permit, University of Windsor: AUPP – Reproductive Strategies of Arctic-Breeding Common Eiders 19–11.

Drone operations in this study were performed in accordance with the rules of the Canadian Aviation Regulations and Nunavut Wildlife Research Permit WL-2019-027, and the pilot obtained a Drone Pilot Certificate from Transport Canada (Issued: 2019-02-28, certificate number: PC1905952549, Transport Canada Account Number: TC1905980118). The DJI Phantom 4 Pro was registered with Transport Canada on 2019-05-23 to Christopher Harris.

## Supplementary Information

Supplementary Information 1.

Supplementary Information 2.

## Data Availability

The datasets used and/or analysed during the current study available from the corresponding author on reasonable request.
