# Supplementary material for: A colonial-nesting seabird shows no heart-rate response to drone-based population surveys
Source: Sci Rep. 2022 Nov 5;12:18804. doi: 10.1038/s41598-022-22492-7 (PMC9637139; doi:10.1038/s41598-022-22492-7)
Supplement: Supplementary file 1 — Supplementary Information 1. [file 41598_2022_22492_MOESM1_ESM.pdf]

Additional tables for “A colonial-nesting seabird shows no heart-rate response to drone-based population surveys”

Geldart et al. (2022)

Table S2 Number of heart-rate samples collected for each Common eider (*Somateria mollissima*) in each drone distance category during drone surveys on June 29th 2019.

**Distance Category**	**Eider ID**										
	C18	*C20*	*C23*	*C24*	*C25*	*C26*	*C31*	*E08*	*E19*	*E20*	*E27*
Control	3	3	3	3	2	3	3	3	3	3	3
0-150m	2	2	1	2	0	2	0	1	3	1	0
151-300m	3	2	3	2	0	3	0	3	3	1	3
>300m	2	1	3	3	0	3	1	3	3	3	2
