# Supplementary material for: A colonial-nesting seabird shows no heart-rate response to drone-based population surveys
Source: Sci Rep. 2022 Nov 5;12:18804. doi: 10.1038/s41598-022-22492-7 (PMC9637139; doi:10.1038/s41598-022-22492-7)
Supplement: Supplementary file 2 — Supplementary Information 2. [file 41598_2022_22492_MOESM2_ESM.pdf]

Drone Reporting Protocol for "A colonial-nesting seabird shows no heart-rate response to drone-based population surveys"

Produced by Andrew Barnas and Christopher Harris, July 2020

1. Project Overview

The objective of this research was to examine the physiological impact of multi-rotor drone surveys on nesting Common eiders (*Somateria mollissima*, hereafter "eiders"). During drone surveys intended to map nest locations on Mitivik Island, Nunavut, we examined the change in heart rate of 11 female eiders using heart-rate monitors deployed within eider nest bowls. For each bird, data was collected during a reference period without drone flights and compared to time periods during drone surveys when the drone was at different distances from the nest location (see main manuscript text for details). For the purposes of this study we did not use any imagery collected from the drone, but rather report details on the characteristics of the aircraft and survey patterns. Therefore, we do not discuss details of the drone's sensors beyond a demonstration of image quality (see section *3.1 Data overview*).

2. Drone System and Operation Details

2.1 Platform specifications

Drone surveys were performed with a DJI Phantom 4 Pro (Dà-Jiāng Innovations, Nanshan, Shenzhen, China), a frequently used multi-rotor drone in wildlife sciences (Figure S1). Specifications of the drone used are as follows: propellers: 4, colour: white, diagonal size (without propellers): 35 cm, weight: 1388 g, flight speed: approximately 10m/s, power source: one removable lithium polymer battery (15.2 V, 5870 mAh), estimated endurance: 30 mins, operational temperature range: 0 – 40 °C, max wind speed resistance: 10 m/s. For manufacturer specifications on latest available models, see www.dji.com/ca/phantom-4-pro (accessed July 23rd 2020).

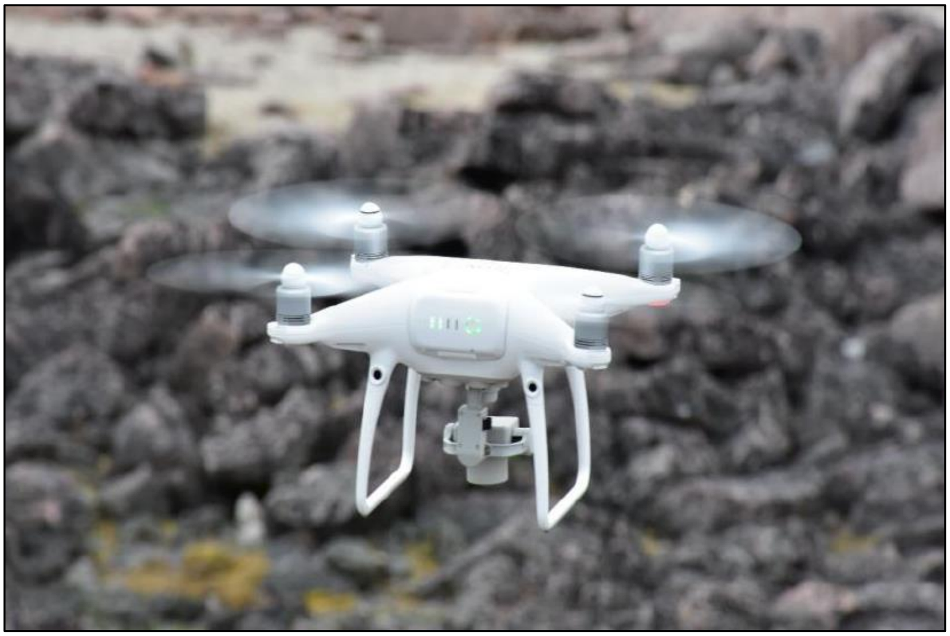

Figure S1 The DJI Phantom 4 Pro used for eider surveys on Mitivik Island in June 2019. Photo credit: Dr. Evan Richardson.

2.2 Takeoff and Retrieval

Drone flights were initiated within the Mitivik Island camp compound, situated behind an electric fence to protect researchers from polar bears (*Ursus maritimus*). No specialized takeoff or retrieval equipment was required for the DJI Phantom 4 Pro, as these models exhibit vertical takeoff and landing procedures. However, a small sheet of plywood was used as a launch and landing pad to facilitate both processes. Prior to takeoff, the pilot performed a pre-flight checklist and uploaded the flight plan to the drone. After takeoff, the drone moved towards the first point of the survey and began automatically flying tracks (*see 2.3 Flight planning and method of operation*). Upon the completion of survey, the drone would automatically return to the landing area, and the pilot typically took manual control of the aircraft to ensure a safe landing (rather than rely on automated landing procedures). Flight data was downloaded from the onboard SD cards after each flight.

2.3 Flight planning and method of operation

The drone flights used in this study were originally intended for the mapping of common eider nest locations on Mitivik Island. Flights were planned as individual line transects semi-autonomously flown by the drone, designed to capture and map an area of interest (Figure S2). Flights were planned using Pix4Dcapture v4.4.0 (Pix4D, Switzerland), where users specify takeoff and landing locations, as well as the desired degree of image overlap for each survey. All flights on June 29th had 30% forward and side image overlap.

Following takeoff, the drone automatically flew towards the starting point for the survey. From there, the drone would automatically follow each transect, collecting images along the transect at the rate specified to achieve the desired image overlap. Upon completion of a transect, the drone would automatically continue with the next transect. Live monitoring of the drone during flight was done by the pilot using an iPad (Apple, California, United States) with the Pix4Dcapture app. The pilot could monitor several aspects of the drone including: the battery status, number of images acquired, and location. While the drone executed flight plans automatically, the pilot could intervene at any time and control the drone manually (e.g. loss of satellite signal).

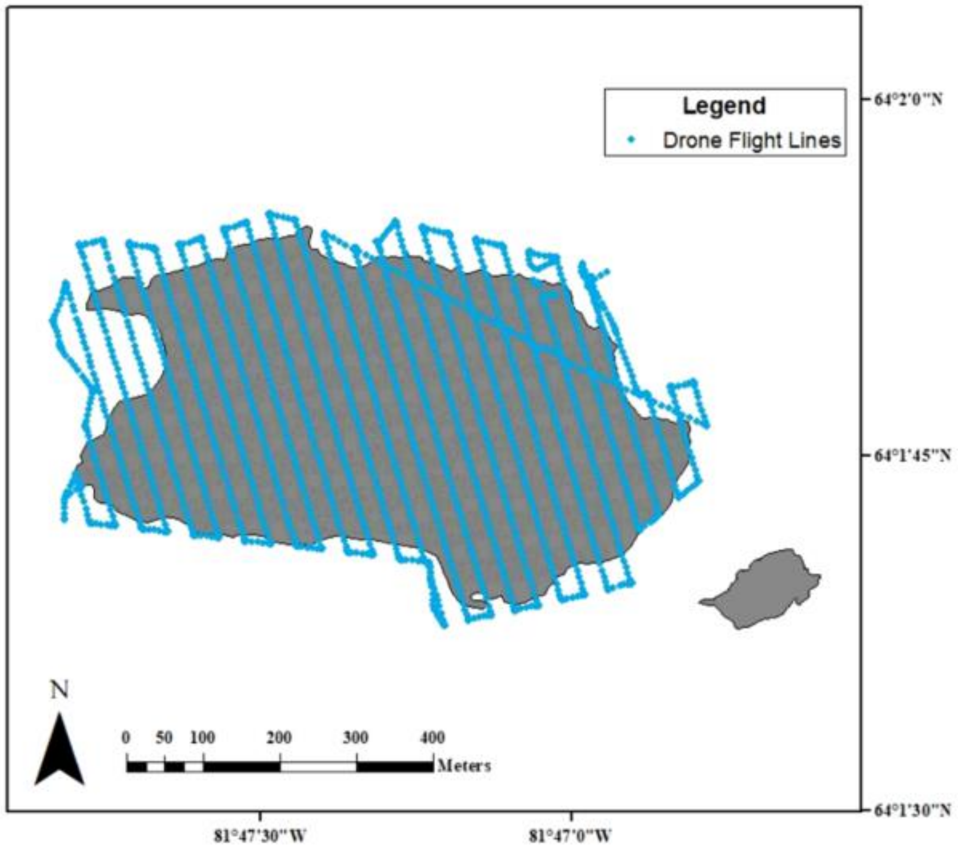

Figure S2 Flight lines of the DJI Phantom 4 Pro across Mitivik Island during eider nesting survey flights on June 29th 2019. Figure created in ArcMap 10.6.1 (https://www.esri.com/en-us/arcgis/products/arcgis-desktop/resources).

3. Payload, sensor, and data collection

3.1 Data overview

While drone flights were originally planned with the objective of mapping eider nest locations, the purpose of this study was to evaluate the physiological impact of these drone surveys on individual eiders. No imagery/sensor data collected from the drone was used in this manuscript, with the exception of the drone's GPS location during flight. However, to demonstrate that RGB imagery collected at the survey altitude flown is adequate for locating eider nests, we provide example raw imagery (with annotated example nest locations) in Figure S3.

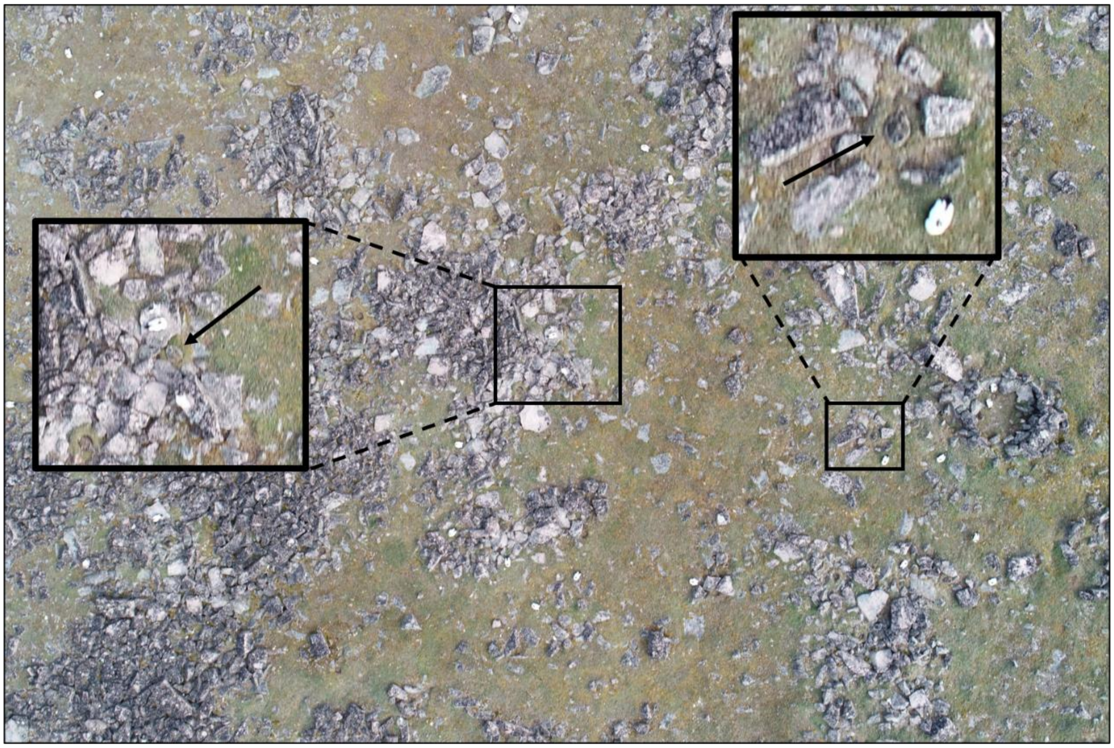

Figure S3 Example raw RGB imagery of nesting eiders collected from the DJI Phantom 4 Pro. Image taken at 30m Above Ground Level (AGL), June 29th 2019.

3.2 Payload or sensor description and data collection methods

We intentionally do not discuss details imagery sensor details here, since no imagery was used for the purposes of this manuscript. The only data from the drone used in this study was the real-time GPS location recorded by the drone during flights. Location information from the DJI Phantom 4 Pro is highly precise, GPS hover accuracy according to manufacturer specifications: vertical ± 0.5m , horizontal ± 1.5 m. The drone collected 21,480 GPS points during flights on June 29th. Points were collected at a mean rate of 10.2 points each second (standard deviation = 0.66).

4. Field Operation Details

All flights in this study were done using the DJI Phantom 4 Pro. Our island survey of nesting eiders was done on June 29th, 2019, but the survey consisted of two individual flights (Table S1). This was done since a survey of the entire study area could not be done in a single flight due to battery limitations. Surveys were flown at 30m Above Ground Level (AGL).

Table S1. Summary of drone flights on June 29th, 2019, with start and end times of individual flights.

Survey Date	Flight Altitude (m AGL)	Flight Number	Start Time	End Time
June 29th ,2019	30	1	08:52:37	09:13:12
June 29th ,2019	30	2	09:17:28	09:35:59

5. Data post-processing

The DJI Phantom 4 Pro’s flight logs recorded GPS locations and associated these with a Unix timestamp. To facilitate user interpretation, we converted these timestamps to a Year-Month-Day Hour:Minute:Second format using package *lubridate* in R (Grolemund and Wickham 2011). Since multiple GPS points were collected each second by the drone during flight, we filtered the data to only include the first observation collected each second. These locations were used to estimate the distance of the drone to each eider nest at a given time during the survey (see main manuscript text).

6. Permits, regulations, training, and logistics

Drone operations in this study were performed in accordance with the rules of the Canadian Aviation Regulations, Nunavut Wildlife Research Permit WL-2019-027, and the pilot obtained a Drone Pilot Certificate from Transport Canada on. The DJI Phantom 4 Pro was registered with Transport Canada on 2019-05-23 to Christopher Harris (University of Windsor), and marked with a registration number prior to flight operations.

The main limitation of the drone used in this study was the relatively short flight times While a single survey of the island is planned as one set of flight lines, these surveys often consisted of several distinct flights. If the battery level fell below approximately 30%, the pilot would pause the current flight and bring the drone back to the landing zone for a battery replacement. Once the battery had been replaced and the drone could continue its flight by returning to the point that it had previously paused the mission.

References

Grolemund, G., & Wickham, H. (2011). Dates and times made easy with lubridate. *Journal of Statistical Software*, *40*(3), 1-25.
